# Nurses’ burnout and quality of life: A systematic review and critical analysis of measures used

**DOI:** 10.1002/nop2.936

**Published:** 2021-05-15

**Authors:** Haitham Khatatbeh, Annamária Pakai, Tariq Al‐Dwaikat, David Onchonga, Faten Amer, Viktória Prémusz, András Oláh

**Affiliations:** ^1^ Doctoral School of Health Sciences Faculty of Health Sciences University of Pécs Pécs Hungary; ^2^ Institute of Nursing Sciences Basic Health Sciences and Health Visiting Faculty of Health Sciences University of Pécs Pécs Hungary; ^3^ Jordan University of Science and Technology Ar‐Ramtha Jordanz

**Keywords:** burnout, nurses, quality of life

## Abstract

**Background:**

Nurses’ burnout might affect their quality of life, productivity and nursing care services.

**Aim:**

The aim of this systematic review was to systemically review the relationship between nurses’ burnout and quality of life and to introduce practical recommendations to reduce nurses’ BO and improve their QOL.

**Methods:**

In April 2021, MeSH terms (("Nurses"[Mesh]) AND "Burnout, Professional"[Mesh]) AND "Quality of Life"[Majr] were used to search five electronic databases: CINAHL, PubMed, Medline, Psychology and Behavioral Sciences Collection and Google Scholar.

**Results:**

The search produced 21 studies exploring nurses’ burnout and their quality of life within the last ten years (2009–2021). Most of these studies found significant relationships between the burnout dimension(s) and quality of life dimension(s) among the nurses.

**Conclusion:**

Nurses have moderate to high levels of burnout and were negatively associated with poor quality of life. Interventional programs are needed to decrease nurses’ burnout and improve their quality of life.

## 1 INTRODUCTION

Burnout (BO) is attracting considerable attention due to its serious consequences, whether on staff productivity, client satisfaction or institutions’ reputation (Manzano‐García & Ayala, 2017; Maslach et al., 1986). BO also has several physical effects, such as musculoskeletal diseases, mental effects such as depression and job‐related effects such as absenteeism (Salvagioni et al., 2017).

It is well known that the nurses are among those staff dealing and working with many people, including patients, families and other co‐workers, which make them vulnerable to BO. (Chou et al., 2014; Gómez‐Urquiza et al., 2017; Manzano‐García & Ayala, 2017; Messias et al., 2019). The possible reasons that make nurses particularly vulnerable to BO might include the extra time needed to follow‐up patients and families’ requests, lack of respect, teamwork and collaboration between nurses and other healthcare professionals, and nurses’ poor coping skills to deal with these stressors.

In addition to other factors such as poor work environment, high workload and low salaries, BO might affect nurses’ Quality of Life (QOL) (Naz et al., 2016). Furthermore, nurses’ BO might also increase absenteeism and affect their QOL (Aytekin et al., 2013; Wu et al., 2011). Nurses’ absenteeism and low QOL might ultimately affect the patient safety and quality of nursing care provided to patients (Kelleci et al., 2011). So, BO and its consequences might affect nurses’ QOL (Aytekin et al., 2013; Azari & Rasouyar, 2016; Hatamipour et al., 2017).

Nurses’ QOL is also getting more attention because they are prone to physical, psychological and social stressors (Serinkan & Kaymakçi, 2013). Many researchers have systematically reviewed BO in paediatric, gynaecology, emergency and primary nursing (De La Fuente‐Solana et al., 2019; Gómez‐Urquiza et al., 2017; Monsalve Reyes et al., 2018; Pradas‐Hernández et al., 2018), and another researcher has systematically reviewed BO associations with social support (Velando‐Soriano et al., 2020). However, none of these systematic reviews has examined the relationship between nurses’ BO and their QOL.

### 1.1 Definitions of BO and QOL

According to Maslach et al., (1986), BO is a syndrome of combined emotional exhaustion, depersonalization and reduced personal accomplishment. Emotional exhaustion entails a psychological feeling of being unable to give because of depleted emotional resources (Maslach et al., 1986). In depersonalization, the staff becomes unfeeling or hard‐hearted with clients (Maslach et al., 1986). The reduced personal accomplishment is to be dissatisfied about own job accomplishments (Maslach et al., 1986).

Similarly, World Health Organization (WHO) described BO as a syndrome of exhaustion, feeling of negativism and decreased personal efficacy due to long‐lasting work stress that was not effectively treated (World Health Organization, 2018). On the other hand, Kristensen et al., (2005) described BO’s essence as fatigue and exhaustion, which attribute to different domains in the person's life. Also, the *Conversation of Resources theory* was used in defining BO as a feeling of emotional exhaustion, physical fatigue and cognitive weariness (Schilling et al., 2019; Shirom, 2004).

BO definitions were different from each other; each definition included a set of BO components. For example, the definition of Maslach et al., (1986) had emotional exhaustion, depersonalization and reduced personal accomplishment. In the Shirom–Melamed definition, the components were different: emotional exhaustion, physical fatigue and cognitive weariness (Schilling et al., 2019; Shirom, 2004). On the other hand, the WHO definition included exhaustion, negativism and decreased personal efficacy (World Health Organization, 2018).

QOL is a general and relatively new expression that replaced old words like happiness and well‐being (Serinkan & Kaymakçi, 2013). QOL is defined by WHO as a humans’ impression about their situation in life within their environment regarding their aims, values, prospects and worries (WHO, 1997). Professional QOL (ProQOL) is a subtype of the QOL for helping others overcome their suffering and trauma (Stamm, 2010).

The WHO definition was very comprehensive and related to general health (WHO, 1997). On the other hand, the definition of professional QOL is related to work‐related QOL. However, the definition of professional QOL is very comprehensive regarding the work environment (Stamm, 2010).

### 1.2 Measures of BO and QOL

The Maslach Burnout Inventory (MBI) is the most widely used instrument to measure the individual's experience of BO (Kristensen et al., 2005). It measures the three aspects of BO syndrome, namely emotional exhaustion, depersonalization and personal accomplishment (Kristensen et al., 2005). The MBI is composed of 16–22 Likert‐type items depending on the used version, general, human services, students, medical personnel or educators’ version (Maslach et al., 1986).

The Copenhagen Burnout Inventory (CBI) is another valid instrument to measure BO (Kristensen et al., 2005). It was developed as a part of the Danish Project on BO, Motivation and Job Satisfaction (Borritz et al., 2006; Kristensen et al., 2005). The CBI is composed of 19 Likert‐type items to measure three dimensions of BO: personal BO, work‐related BO and client‐related BO among professionals who work with clients (Kristensen et al., 2005).

The Oldenburg Burnout Inventory (OLBI) is another valid instrument used to measure BO among the various professionals using 16 Likert‐type items (Janko & Smeds, 2019; Reis et al., 2015). Like MBI, the OLBI measures BO as a syndrome but encompasses only two dimensions: exhaustion and disengagement from work (Reis et al., 2015).

The Shirom–Melamed Burnout Questionnaire (SMBQ) is composed of twelve items to measure BO’s three dimensions, namely emotional exhaustion, physical fatigue and cognitive worn‐out, as‐built according to *Conversation of Resources theory* (Schilling et al., 2019).

Although MBI is considered the golden instrument in measuring BO, Kristensen et al., (2005) criticized the MBI because it measures the three dimensions of BO syndrome independently. This conflicts with Maslach's definition that the three dimensions of BO co‐occur (Kristensen et al., 2005). On the other hand, it is unnecessary to use the three CBI subscales to measure the BO (Kristensen et al., 2005). Depending on the target population, only one or two subscales of the CBI can be used (Kristensen et al., 2005). The CBI was translated into other languages and found to have acceptable validity and reliability (Berat et al., 2016; Chin et al., 2018; Fiorilli et al., 2015; Kristensen et al., 2005; Mahmoudi et al., 2017; Yeh et al., 2007).

WHO developed one of the most important tools to measure QOL (WHOQOL). WHOQOL comprises 100 Likert‐type items covering six main areas: physical health, psychological health, social relationships, and environment, the level of independence and spirituality (WHOQOL‐Group, 1998). The short version of WHOQOL is WHOQOL‐BREF, which comprises 26 Likert‐type items that cover four main areas: physical health, psychological health, social relationships and environment (WHOQOL‐Group, 1998).

The Short‐Form Health Survey (SF‐36) is another tool to assess QOL. SF‐36 is composed of 36‐items measuring different health domains: physical and psychological (Ware & Sherbourne, 1992). The physical health domains in SF‐36 are physical working, physical role, pain and overall health (Ware & Sherbourne, 1992). On the other hand, the mental health domains in SF‐36 are vitality, social functioning, emotional role and psychological health (Ware & Sherbourne, 1992). SF‐36 was further shortened into SF‐12, measuring only two dimensions physical and mental component (Ware et al., 1994).

ProQOL tool is composed of 30 Likert‐type items to assess QOL (Stamm, 2010). ProQOL measures both positive and negative consequences of dealing with humans suffering from traumatic situations (Stamm, 2010). ProQOL measures *Compassion Satisfaction* and *Compassion Fatigue*, which is composed of BO and Secondary Traumatic Stress (Stamm, 2010). *Compassion Satisfaction* is to like and be happy doing your job tasks effectively (Stamm, 2010). As a *Compassion Fatigue* sub‐domain, BO was described as a feeling of hopelessness and problems dealing with work or doing your tasks well (Stamm, 2010). Secondary Traumatic Stress is related to job nature and interaction with persons complaining of severe stressful situations (Stamm, 2010).

Although WHOQOL, SF‐36 and ProQOL are the most widely used tools to measure QOL, some researchers used other validated tools. For instance, the Work‐Related Quality of Life Scale (WR‐QOLS) is another validated questionnaire measuring QOL. WR‐QOLS assesses six dimensions of QOL: general well‐being, home‐work interface, job and career satisfaction, control at work, working conditions and stress at work (Casida et al., 2019; Wang et al., 2019). WR‐QOLS comprises 23 items of 5‐point Likert‐type scale ranging from strongly disagree to strongly agree (Casida et al., 2019; Wang et al., 2019). Additionally, Work‐Life Quality (QWL) encompasses 35‐items measuring eight dimensions of work‐related QOL (Permarupan et al., 2020). Last, another QOL scale comprises 28‐items assessing four dimensions: working life, social life, BO and satisfaction (Çelmeçe & Menekay, 2020).

### 1.3 Purpose

The purpose of this systematic review is to examine the relationship between nurses’ BO and their QOL based on the existing research. The objectives of this review include describing nurses’ BO and how it was measured, describing nurses’ QOL and how it was measured, assessing the relationship between nurses’ BO and their QOL, and introducing practical recommendations to reduce nurses’ BO and improve their QOL.

## 2 METHODS

PRISMA guidelines were followed to perform this systematic review (Liberati et al., 2009). PRISMA includes evidence‐based items for reporting systematic reviews and meta‐analyses (Liberati et al., 2009). PRISMA illustrates how researchers can ensure the objective and complete reporting of systematic reviews and meta‐analyses (Liberati et al., 2009).

### 2.1 Search strategy

Five electronic databases, CINAHL, PubMed, Medline, Psychology and Behavioral Sciences Collection and Google Scholar, were selected for this systematic review. These databases were selected because they include bibliographic information for articles covering our research topic: nursing and psychology. Two members of the review team searched the chosen databases in April 2021. First, the terms “nurses AND burnout AND quality of life” were used to find the MeSH terms on PubMed. The command line used in searching PubMed was (("Nurses"[Mesh]) AND "Burnout, Professional"[Mesh]) AND "Quality of Life"[Majr]. Searching restrictions included English language, scholarly journals and last twelve years publications (2009–2021).

### 2.2 Study selection

**FIGURE 1:**
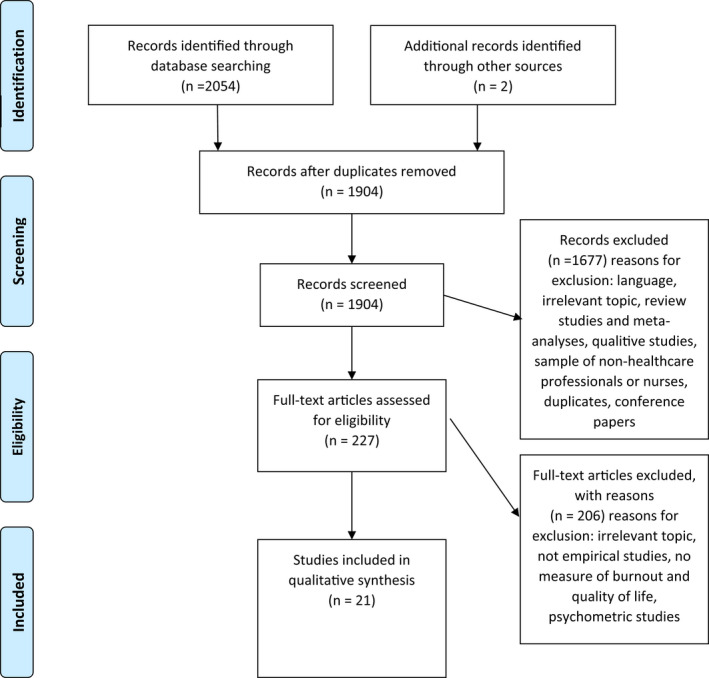
PRISMA flow diagram

To ensure the reliability of the study selection process, it was independently done by two members of the review team. The selection process started by screening titles and abstracts, followed by full reading for the initially selected studies. The chosen studies meeting the inclusion criteria were finally assessed for possible methodological bias using Ciaponni's critical reading checklist. To resolve any disagreement, a third member of the review team was consulted. See PRISMA flow diagram, Figure 1.

### 2.3 Quality assessment

According to similar systematic reviews (De La Fuente‐Solana et al., 2019; Gómez‐Urquiza et al., 2017; Monsalve Reyes et al., 2018) and because all of the included articles were cross‐sectional studies, items related to internal validity (2, 3, 4,5, 6, 11, 12, 13, 14‐b, 15, 16, 17 and 18) from Ciaponni's critical reading checklist (Ciapponi, 2010) were used to assess the quality of the articles. Each article was assessed for methodological quality, and no one was excluded for a methodological bias. The results of critical reading are shown in Additional file 1.

### 2.4 Inclusion criteria

According to the predetermined inclusion criteria for this systematic review, we included only the cross‐sectional and peer‐reviewed studies measuring both nurses’ BO and QOL using separate validated measures. The exclusion criteria were as follows: (a) studies that didn't measure both BO and QOL, (b) studies that did not specify proportion or number of the nurses, (c) psychometric studies, (d) qualitative studies as they do not provide numerical measurements BO and QOL, (e) interventional studies, (f) preprints and (g) studies with other languages.

### 2.5 Data extraction

After applying the predetermined inclusion and exclusion criteria for the search performed in April 2021, 21 studies were included in this systematic review (Figure 1). Most of the studies (*n* = 12) included were from China, Turkey, Iran, Greece or Pakistan (Aytekin et al., 2013; Azari & Rasouyar, 2016; Çelmeçe & Menekay, 2020; Erkorkmaz et al., 2018; Fradelos et al., 2014; Hatamipour et al., 2017; Kelleci et al., 2011; Naz et al., 2016; Paniora et al., 2017; Wang et al., 2019; Wu et al., 2011; Zeng et al., 2020). The rest of the studies was from Brazil (Ribeiro et al., 2021), Egypt (Abdel‐Aziz & Adam, 2020), Korea (Kim et al., 2019), India (Abraham & D’silva, 2013), Jordan (Khatatbeh et al., 2020), Malaysia (Permarupan et al., 2020), Poland (Kupcewicz & Jóźwik, 2020), Saudi Arabia (Alotni & Elgazzar, 2020) and the USA (Casida et al., 2019). All of the studies included in this systematic review utilized a cross‐sectional design, and most of them (*n* = 11) were published between 2019 and 2021, Table 1.

**TABLE 1:** Results of searching electronic databases

Database	Articles found
CINAHL	1,364
PubMed	142
Medline	402
Psychology and Behavioral Sciences Collection	108
Google Scholar	38
Total	2054

For the 21 included articles, the following information was independently extracted by two researchers: (a) the first author's surname, (b) year of publication, (c) research design, (d) sampling method and size, (e) BO instrument, (f) QOL instrument and (g) results. If there was a disagreement about a certain article, a third member of the research team was consulted until an agreement was reached.

## 3 RESULTS

### 3.1 Summary of the reviewed studies

The total number of nurses in the 21 included studies was 9,859. Regarding the gender of participants, three studies surveyed only female nurses (Azari & Rasouyar, 2016; Naz et al., 2016; Wu et al., 2011). Concerning the profession of participants, one of these studies compared female nurses to female doctors (Wu et al., 2011), another study compared nurses to nurse educators (Abraham & D’silva, 2013), and one study studied different healthcare providers, including nurses (Çelmeçe & Menekay, 2020). Regarding the working area of the participants, four studies surveyed mental nurses (Abdel‐Aziz & Adam, 2020; Fradelos et al., 2014; Paniora et al., 2017; Zeng et al., 2020), one study surveyed only Neonatal Intensive Care Unit (NICU) nurses (Aytekin et al., 2013), one study surveyed paediatric nurses (Khatatbeh et al., 2020), one study surveyed emergency nurses (Ribeiro et al., 2021) and one study included nurses working at critical care units (Alotni & Elgazzar, 2020) (Table 2). Also, one study surveyed nurses caring for COVID‐19 patients (Çelmeçe & Menekay, 2020).

**TABLE 2:** Summary of the included studies

	Author, Year, Country	Study Design	Sampling method & size	BO instrument	QOL instrument	Main results
1.	Abdel‐Aziz and Adam, (2020), Egypt	Descriptive, Correlational	A purposive sample, 100 psychiatric nurses	MBI−22 items	ProQOL−30 items	A high significant relationship was found between nurses’ BO and their professional QOL
2.	Abraham and D’silva, (2013), India	Descriptive, Cross‐Sectional	Random sampling, 50 nurses	Shirom–Melamed BO inventory	WHOQOL‐BREF (26 items)	Significant weak negative correlation between BO and both psychological QOL and social QOL
3.	Alotni and Elgazzar, (2020), Saudi Arabia	Descriptive, Correlational	A purposive sample, 170 critical care nurses	MBI−22 items	Short‐Form Health Survey −12 (SF12)	Burnout and quality of life are significantly and negatively correlated
4.	Aytekin et al. (2013), Turkey	Descriptive, Correlational	2 hospitals total population sampling, 80 NICU nurses	MBI−22 items	WHOQOL‐BREF (26 items)	As BO level increased, the QOL of the nurses decreased
5.	Azari and Rasouyar, (2016), Iran	Descriptive, Correlational	Simple random sampling, 150 Female nurses	MBI−22 items	Short‐Form Health Survey −36 (SF36)	Significant correlation between QOL and its components, with of job BO
6.	Casida et al., (2019), USA	Exploratory, correlational	A random sample, 104 nurse practitioners	CBI−19 items	WR‐QOLS 23 items	A negative correlation found between work‐related burnout and quality of work‐life
7.	Çelmeçe and Menekay, (2020), Pakistan	Descriptive, Cross‐Sectional	Convenient sample 120 nurses	MBI−22 items	Menekay & Çelmeçe QOL 28‐items	Healthcare providers and nurses QOL is affected by burnout.
8.	Erkorkmaz et al., (2018), Turkey	Analytical, Cross‐Sectional	Voluntary sample from one hospital, 131 nurses	MBI−22 items	ProQOL−30 items	Emotional Exhaustion and Personal Accomplishment significantly affected Compassion Satisfaction
9.	Fradelos et al., (2014), Greece	Descriptive, Cross‐Sectional	139 nurses general and mental hospitals	MBI−22 items	Short‐Form Health Survey −36 (SF36)	BO impacts QOL of nurses
10.	Hatamipour et al., (2017), Iran	Descriptive, Cross‐Sectional	Multistage cluster sampling, 400 nurses	MBI−22 items	WHOQOL‐BREF (26 items)	BO had a negative and significant relationship with QOL and Perceived social support
11.	Kelleci et al., (2011), Turkey	Descriptive, Cross‐Sectional	Cluster sampling for 3 hospitals, 439 nurses	MBI−22 items	WHOQOL‐BREF (26 items)	There was a negative relationship between all subdimensions of QOL and exhaustion and desensitization scores
12.	Khatatbeh et al., (2020), Jordan	Descriptive, Cross‐Sectional	Convenient sample 225 paediatric nurses	CBI−19 items	WHOQOL‐BREF (26 items)	The three CBI subscales were negatively correlated with the four QOL subscales
13.	Kim et al., (2019), Korea	Descriptive, Cross‐Sectional	Convenient sample 324 nurses	MBI−22 items	ProQOL−30 items	The three MBI subscales were significantly correlated with the two ProQOL subscales
14.	Kupcewicz and Jóźwik. (2020) Poland	Comparative, Cross‐Sectional	Convenient sample 1806 nurses	CBI−19 items	WHOQOL‐BREF (26 items)	The three CBI subscales were found explaining nurses QOL
15.	Naz et al., (2016), Pakistan	Descriptive, Cross‐Sectional	Convenience sampling, 106 Female nurses	MBI−22 items	WHOQOL‐BREF (26 items)	Nurses’ BO was common because of increasing workload can negatively affect their QOL
16.	Paniora et al., (2017), Greece	Descriptive, Cross‐Sectional	Convenience sampling, 100 mental health nurses	MBI−22 items	Short‐Form Health Survey −36 (SF36)	Psychiatric nurses have low levels of BO. Levels of physical activity are correlated with both QOL and BO syndrome
17.	Permarupan et al., (2020), Malaysia	Descriptive, Cross‐Sectional	Convenience sampling, 432 nurses	MBI−13 items	Walton QWL 35‐items	The psychological empowerment mediates the relationship between quality of work‐life and burnout
18.	Ribeiro et al., (2021), Brazil	Analytical, Cross‐Sectional	Convenience sampling, 83 emergency nurses	MBI−22 items	Short‐Form Health Survey −36 (SF36)	The BO has an influence on nurses’ QOL
19.	Wang et al., (2019), China	Cross‐Sectional, E‐mail‐based survey	multistage stratified cluster random sampling 2,504 nurses	Chinese MBI−15 items	WR‐QOLS 23 items	Job BO has a negative effect on nurses' quality of work‐life
20.	Wu et al., (2011), China	Comparative, Cross‐Sectional	Stratified cluster sampling, 947 female nurses	(MBI‐GS) 16 items	Short‐Form Health Survey −36 (SF36)	Improving nursing working environment is an efficient preventive measure for reducing occupational stress to prevent job BO and improve QOL among female nurses
21.	Zeng et al., (2020), China	Descriptive, Cross‐Sectional	Convenience sampling, 1,449 Psychiatric nurses	Chinese MBI−15 items	WHOQOL‐BREF (26 items)	BO negatively impacted nurses QOL

### 3.2 Definition of BO and QOL in the reviewed studies

The definition of Maslach et al., (1986) was explicitly adopted by five studies (Aytekin et al., 2013; Erkorkmaz et al., 2018; Hatamipour et al., 2017; Wu et al., 2011; Zeng et al., 2020). Six studies implicitly adopted Maslach & Jackson's (1981) definition of BO because they used the MBI without including a BO definition (Abraham & D’silva, 2013; Azari & Rasouyar, 2016; Çelmeçe & Menekay, 2020; Kim et al., 2019; Permarupan et al., 2020; Ribeiro et al., 2021). Two studies adopted the definition of Freudenberger (1974), which described BO as bodily and behavioural signs and symptoms caused by physical and psychological tiredness (Paniora et al., 2017; Wang et al., 2019). One study defined BO as a chronic mental syndrome that results from social stressors (Abdel‐Aziz & Adam, 2020).

QOL was described in three studies as the bodily, psychological and social health interacting with the environment (Aytekin et al., 2013; Erkorkmaz et al., 2018; Paniora et al., 2017). Similarly, other studies (*n* = 3) described QOL as a vital feature of human well‐being established in a bodily, public and community frame (Fradelos et al., 2014; Naz et al., 2016; Wu et al., 2011). Another study by Azari and Rasouyar (2016) described QOL as a multidimensional and multifaceted concept characterized by objective and subjective features and helps finally to assess human well‐being (Azari & Rasouyar, 2016). On the other hand, six studies have adopted the definition of the WHO (Abraham & D’silva, 2013; Alotni & Elgazzar, 2020; Hatamipour et al., 2017; Kelleci et al., 2011; Kupcewicz & Jóźwik, 2020; Ribeiro et al., 2021). Last, some studies examined work‐related or professional QOL, not general QOL (Abdel‐Aziz & Adam, 2020; Casida et al., 2019; Erkorkmaz et al., 2018; Kim et al., 2019).

### 3.3 Measures of BO and QOL used in the reviewed studies

Out of the 21 studies included in this review, 17 studies measured BO using a version of MBI (Abdel‐Aziz & Adam, 2020; Alotni & Elgazzar, 2020; Aytekin et al., 2013; Azari & Rasouyar, 2016; Çelmeçe & Menekay, 2020; Erkorkmaz et al., 2018; Fradelos et al., 2014; Hatamipour et al., 2017; Kelleci et al., 2011; Kim et al., 2019; Naz et al., 2016; Paniora et al., 2017; Permarupan et al., 2020; Ribeiro et al., 2021; Wang et al., 2019; Wu et al., 2011; Zeng et al., 2020). Three studies used the CBI (Casida et al., 2019; Khatatbeh et al., 2020; Kupcewicz & Jóźwik, 2020), and one study used Shirom–Melamed BO inventory (Abraham & D’silva, 2013). To measure nurses’ QOL, the included 21 studies have used either WHOQOL‐BREF (*n* = 8), SF‐36 or SF‐12 (*n* = 6), ProQOL (*n* = 3) or another tool (*n* = 4). Most of the included studies found moderate to high levels of BO. However, psychiatric nurses showed low levels of BO in one study (Paniora et al., 2017).

### 3.4 The relationship between BO and QOL in the reviewed studies

The majority of the studies (*n* = 16) found a negative correlation between nurses’ burnout and their QOL or professional QOL (Abdel‐Aziz & Adam, 2020; Abraham & D’silva, 2013; Alotni & Elgazzar, 2020; Aytekin et al., 2013; Casida et al., 2019; Erkorkmaz et al., 2018; Fradelos et al., 2014; Hatamipour et al., 2017; Kelleci et al., 2011; Khatatbeh et al., 2020; Kim et al., 2019; Kupcewicz & Jóźwik, 2020; Permarupan et al., 2020; Ribeiro et al., 2021; Wang et al., 2019; Zeng et al., 2020). For example, nurses’ QOL was negatively correlated with emotional exhaustion and depersonalization, and positively with personal accomplishment (Kelleci et al., 2011). Similarly, the emotional exhaustion among NICU nurses was negatively associated with all QOL subscales; and depersonalization was negatively associated with physical, psychological health and social relationships subscales (Aytekin et al., 2013). Two domains of QOL, psychological and social relationships, were negatively correlated with BO (Abraham & D’silva, 2013). Similarly, another study found that personal accomplishment affects nurses’ QOL (Erkorkmaz et al., 2018). One study found a significant correlation between emotional exhaustion and QOL measured by SF‐36 (Azari & Rasouyar, 2016). An intermediate effect was found for emotional exhaustion on Compassion Fatigue, the subscale of ProQOL (Erkorkmaz et al., 2018). Similar results were found between the depersonalization subscale and two subscales of ProQOL: BO and Compassion Fatigue (Erkorkmaz et al., 2018). Another study found a strong negative correlation between both emotional exhaustion and depersonalization with nurses’ QOL (Fradelos et al., 2014). Also, some studies (*n* = 4) found that professional or work‐related QOL was also negatively associated with nurses’ BO (Abdel‐Aziz & Adam, 2020; Casida et al., 2019; Kim et al., 2019; Wang et al., 2019).

## 4 DISCUSSION

Assessment of nurses’ BO, their QOL, and the relationship between BO and QOL were the aims of this systematic review. The high levels of nurses’ BO in the reviewed articles were explained by the challenging work conditions and working environments such as changing shifts, low nurse‐to‐patient ratio, and poor teamwork and collaboration with other healthcare workers (Erkorkmaz et al., 2018). However, the varying levels of BO across the included studies can be explained by the various working environments such as unit/ward, the different working shifts and the different working loads. For example, some studies studied only NICU, mental, critical or paediatric nurses; and some studies included only one or two hospitals in their studies. The NICU’s busy environment, the critical patients’ cases, ventilator sounds and cardiac monitor alarms might make the nurses more susceptible to BO than those in other units. Furthermore, the nurses who work on the night or alternate shifts and the associated sleep problems might have higher BO than other nurses who work on the day and regular shifts. For instance, the low BO levels found among psychiatric nurses in the study of Paniora et al., (2017) might not be generalizable to all nurses because of the low sample size. However, this finding is relatively consistent with a study that revealed low to moderate scores on MBI subscales (Kilfedder et al., 2001). On the other hand, this result is different from a previous study that showed moderate to high scores on MBI subscales (Hamaideh, 2011).

Most of the included studies have explicitly concluded that nurses’ BO or its’ subscales negatively impacts their QOL or its’ subscales (Abraham & D’silva, 2013; Alotni & Elgazzar, 2020; Aytekin et al., 2013; Fradelos et al., 2014; Hatamipour et al., 2017; Kelleci et al., 2011; Khatatbeh et al., 2020; Kupcewicz & Jóźwik, 2020; Ribeiro et al., 2021; Zeng et al., 2020). Similarly, some of the included studies found a negative association between professional or work‐related QOL and nurses’ BO (Abdel‐Aziz & Adam, 2020; Casida et al., 2019; Erkorkmaz et al., 2018; Kim et al., 2019; Permarupan et al., 2020; Wang et al., 2019). Although some studies did not find a significant correlation between nurses’ BO and QOL, they found moderate to high levels of BO and relatively poor QOL (Kupcewicz & Jóźwik, 2020; Naz et al., 2016; Paniora et al., 2017; Permarupan et al., 2020; Wu et al., 2011).

In the study of Kelleci et al., (2011), the negative relationship between nurses’ BO and their QOL was explained by their low job satisfaction. In the study of Aytekin et al. (2013), the moderate levels of nurses’ BO impacting their QOL might be explained by NICU’s environment and high workload. The low personal accomplishment scores and their relationship with QOL, in the study of Erkorkmaz et al., (2018), were explained by the high occupational stress.

Due to their impact on nurses’ health and patient care, comprehensive interventional programs such as salary increment, decreasing the working hours and counselling sessions on stress management are needed to prevent nurses’ BO and improve their QOL. Moreover, social and manager supports are also essential to prevent nurses’ BO and improve their QOL (Hamaideh, 2011), improving the patient safety and quality of nursing care provided to their patients (Khatatbeh et al., 2020). Furthermore, it is essential to control the reasons that initially make nurses susceptible to BO, such as high workload and low satisfaction (Van Bogaert et al., 2013). Traditional and social media can be utilized in showing the bright sides of the nursing profession to enhance respect for nurses, improving the teamwork and collaboration between nurses and other healthcare professionals, and teaching nurses the necessary coping skills and strategies to deal with stressors.

Our systematic review suggests that nurses are complaining of moderate to high levels of BO. Also, the high levels of BO among nurses are negatively associated with low QOL. So, nurses’ BO needs to be controlled because it might affect their QOL and the quality of nursing care. Many possible measures that might decrease nurses’ BO and improve their QOL, such as manager support (Khatatbeh et al., 2020), counselling sessions and monetary bonuses. Other targeted interventions might be helpful in addressing the sociodemographic factors such as gender, unit and shift that were found to be associated with higher levels of BO and/or lower QOL scores. For instance, female nurses who are married or having families to care for should get more off days, nurses working in critical care units should be assigned to fewer cases, and nurses who work on alternate shifts should get more off days or longer break times.

### 4.1 Limitations

A key problem with some of the studies included in this systematic review is the small sample sizes (Abdel‐Aziz & Adam, 2020; Abraham & D’silva, 2013; Alotni & Elgazzar, 2020; Aytekin et al., 2013; Azari & Rasouyar, 2016; Casida et al., 2019; Çelmeçe & Menekay, 2020; Erkorkmaz et al., 2018; Fradelos et al., 2014; Naz et al., 2016; Paniora et al., 2017; Ribeiro et al., 2021). Moreover, three studies selected nurses from only one or two hospitals (Aytekin et al., 2013; Erkorkmaz et al., 2018; Wu et al., 2011). Additionally, three studies (Alotni & Elgazzar, 2020; Aytekin et al., 2013; Ribeiro et al., 2021) have studied nurses working at critical care units, who have more stressful environment than other nurses. This systematic review might also be limited by including only those studies in English and excluding qualitative studies. Last, the different tools used in the included studies to measure BO and QOL might be another limitation. Future systematic reviews are encouraged to have meta‐analysis by including studies using the same measures. However, the studies included in this systematic review were peer‐reviewed, were done in different countries and continents, and included nurses working in different working areas.

## 5 CONCLUSION

This systematic review aimed to assess the relationship between nurses’ BO and QOL and analyse the measures used. The review results showed moderate to high levels of BO across the included studies, varying levels of QOL and negative relationships between BO and QOL. MBI remains the most widely used instrument in assessing nurses’ BO. Both WHOQOL‐BREF and SF‐36 are the most used tools in measuring nurses’ QOL.

## CONFLICT OF INTEREST

The authors have no personal or financial concern that might lead to a conflict of interest regarding this research.

## AUTHOR CONTRIBUTIONS

H.K., T.D., A.P. and A.O: Plan and design the systematic review. H.K. and T.D: Search and data extraction. H.K: Paper writing. T.D., V.P., F.A., A.P. and D.O: Paper review. All authors: Responsible for research report and approval of the manuscript submission.

## ETHICAL APPROVAL

As this is a systematic review, no ethical approval was needed.

## PATIENT CONSENT FORM

As this is a systematic review, no patients were involved in this study and no consents were needed.

## Data Availability

The data that support the results of this research are available from the corresponding author upon a reasonable request.
